# Over-expression by degradation rescue of RTKs via cancer-secreted autocrine growth factors: *a Phospho-degron-driven actionable layer of post-translational regulation?*

**DOI:** 10.3389/fonc.2023.1278402

**Published:** 2023-09-26

**Authors:** Pierluigi Scalia, Stephen J. Williams

**Affiliations:** ^1^ Istituto Somatogene per la Oncologia Personalizzata e la Ricerca Onco-Genomica (ISOPROG)-Somatolink Expert-Patients For Patients (EPFP) Research Network, Philadelphia, PA, United States; ^2^ Istituto Somatogene per la Oncologia Personalizzata e la Ricerca Onco-Genomica (ISOPROG)-Somatolink Expert-Patients For Patients (EPFP) Research Network, Caltanissetta, Italy; ^3^ Department of Biology, College of Science and Technology, Temple University, Philadelphia, PA, United States

**Keywords:** RTK, gf, UbE3L, UPS, degron, pDegron, paDegron, piDegron

## Abstract

Recently published work provide the first known evidence of a malignancy-associated regulatory mechanism, functionally connecting a phospho-regulated degron domain embedded in a receptor tyrosine kinase (RTK), with its ectopic expression in cancer, conditional to a specific autocrine growth factor signal. Mechanistically, the growth factor-triggered phosphorylation inhibits the degron domain present in the regulated RTK, blocking access to a specific degradation complex. This ultimately rescues the RTK from rapid ubiquitin-proteasome-system-mediated degradation and, most importantly, causes its cellular overexpression. This mechanism, which has been here assigned the new functional name “*Over-Expression by Degradation Rescue*” (*OEDR*), provides an additional layer and potentially preferential tool for the control of RTKs expression in cancer, in addition to other mechanisms acting at the transcriptional and messenger transcript stabilization levels. We propose this newly defined phosphorylation/ubiquitination switch-dependent signal to bear wider unexploited relevance in cell biology and human pathophysiology. The recently identified mechanism underlying an *OEDR*-regulated RTK is discussed herein in the context of physiological endocrine circuits and cancer.

## 1 Introduction

**Figure 1:**
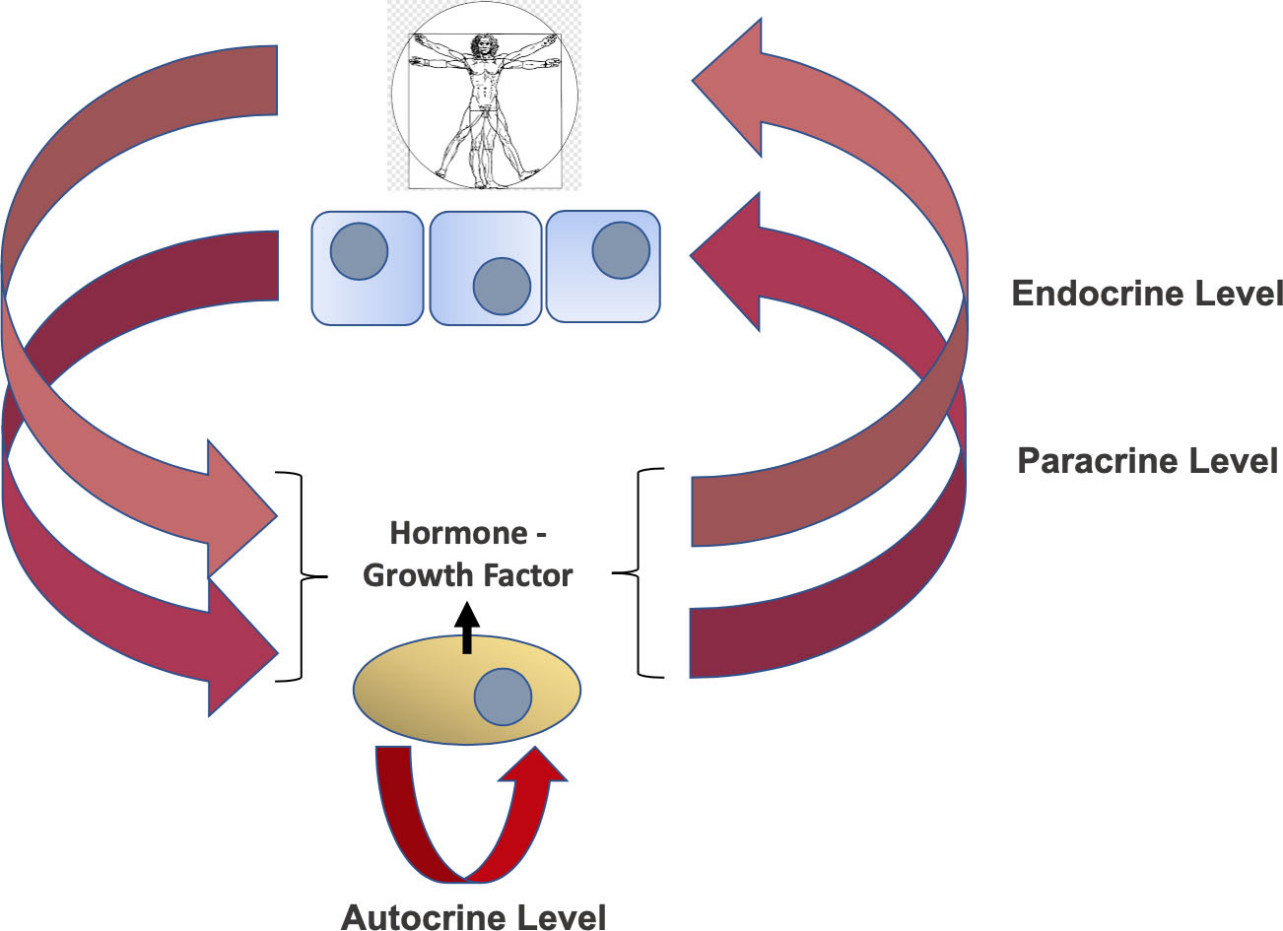
Layers of hormone/growth factor (GF) regulation. Key concept to the mechanism of physiologic and pathologic events regulating hormones and growth factors function is the point of production/secretion (where the hormone/GF-producing cell is located) and the point of specific targeting (where the hormone/GF receptor and transducing effector-bearing cell/tissue is located). In this regard, three layers or modes of action have been described. These layers include the endocrine level (pink arrows), the paracrine level (purple arrows) and the autocrine level (red arrow). The implications of these established regulatory loops with regards to GF-induced *OEDR* of RTKs are discussed in the text.

Three contextual layers of regulation have long been established for the effects of hormones and growth factors in vertebrates and mammalian species. This, depending on the respective localization of the hormone/growth factor producing cell and the target cell bearing its cellular receptor. Specifically, these three layers refer to (a) an endocrine mechanism, when the hormone/GF is produced by a cell/specialized tissue-organ distant from the targeted cell bearing the specific receptor; (b) a paracrine mechanism, when the target cell is in close proximity with the hormone/GF producing cell (as typically observed during developmental tissue growth and in early tumorigenesis); and (c) an autocrine mechanism, when the hormone/GF-secreting cell bears the activated receptor establishing a self-stimulatory loop, as observed in advanced cancer stages (see **Figure 1** for a graphic summary of these three regulatory layers) (1). On a dynamic activation level, these hormonal/GF-generated signals are temporally-regulated in which the duration of each signal depends on the protein expression level/status and consequent availability of the ligand (hormone/growth factor) as well as on its signal transducing receptor at the cellular level. In particular, cellular downregulation and upregulation of hormone/GF receptors are a well-known mechanism for hormone/GF signal self-limiting regulation with mechanistic implications both at the cellular and clinical level. In fact, it has been established that a hormone-GF generated signal and underlying cellular effect following transducing receptor activation tends to temper down within a relatively short time due to the signal-induced parallel downregulation of the stimulated receptor expression, also known as a negative hormone/GF feedback (1). Such acute effect has been associated to ligand-induced subcellular sequestration of the activated trans-membrane receptor through multiple mechanisms, including endocytosis and lysosomal degradation (2, 3). More recently, the advancement in our understanding of ubiquitin-targeted degradation processes has added an additional mechanism responsible for hormone-GF negative feedbacks at the cellular level, namely, the acute and specific RTK targeting by means of ubiquitin-flagging enzymes known as E3 ubiquitin ligases like CBL (reviewed in (4). While this ubiquitin E3 ligase-mediated mechanism has been proposed for RTK downregulation to explain negative hormone-GF feedback loops, no mechanism to date has been suggested for the equally established hormonal-GF positive feedback. Under these circumstances, a hormone-GF initiated stimuli leads to the actual reinforcement of the intracellular signal via upregulation of its transducing receptor(s). The first description of a molecular mechanism which explains such type of positive regulation has come from a study in malignant mesothelioma cell lines establishing a specific functional connection between a a defined autocrine GF-signal and a phospho-inhibited Degron domain embedded in the C-terminal region of an oncogenic RTK (5). More recently, such effect has been further linked to a previously unknown ubiquitin E3 ligase variant (DTX3c) and its associated degradation complex able to acutely clear the cellular RTK expression levels upon autocrine signal block/deprivation (6). As further discussed below, the described intact autocrine GF signal was shown to be critical to maintain high levels of signal transducing RTK in the studied cellular model, fitting with the features of a positive hormone-GF signal. Altogether, the association of a hormonal-GF-dependent negative feedback to a phospho-Degron-bearing RTK, targeted by the CBL ubiquitin E3 ligase (UbE3L) (7), opposed to the recent finding of a positive hormone-GF autocrine signal specifically associated to a phospho-inhibited Degron-bearing RTK, targeted by another UbE3L (DTX3c) (6), allows us to envision a feasible functional context and novel biological scenario for these currently underexploited Degron domains and the underlying phospho-Degron-regulated RTKs. The present opinion/perspective work focuses on the implications of the latter mechanism which we assigned the functional denomination (and acronym) of *Over-Expression by Degradation Rescue* (*OEDR*).

## 2 Over-Expression by Degradation Rescue as a putative mechanism for endocrine-GF positive feedback and RTK cellular upregulation

**Figure 2:**
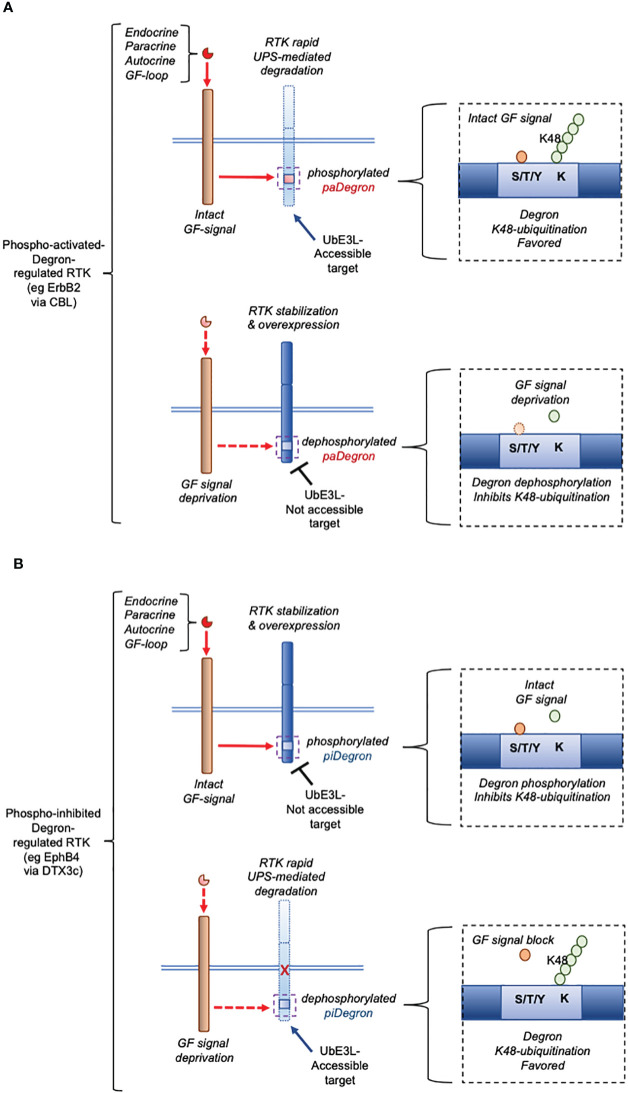
Essential mechanism underlying phospho-activated (*pa*)Degron-mediated expression versus phospho-inhibited (*pi*)Degron-mediated expression of a cellular RTK (graphic summary). **(A)**, pattern of *pa*Degron-mediated RTK expression according to Hormone/GF signal status. The function of a *pa* Degron domain in an RTK is compatible with an endocrine/paracrine negative hormone/GF loop where the signal causes downregulation of the activated (target) receptor (for eg that mediated by CBL) [**(A)** *upper workflow*]. Under reduced hormone/GF levels, the *pa*Degron is generally not made accessible to the UbE3L [**(A)** *lower workflow*]. **(B)**, pattern of *pi*Degron-mediated RTK expression according to Hormone/GF signal status. On the opposite, the *pi*Degron function fits the function of a positive feedback loop [**(B)** *upper workflow*] by extending the RTK expression lifespan and overall protein levels. This type of mechanism is here defined as *Over-Expression by Degradation Rescue* (*OEDR*). Under such circuit, reduced or inhibited autocrine signal leads to rapid clearance of the controlled RTK [**(B)** *lower workflow*]. This type of mechanism (*OEDR*) entails the presence of a *pi*Degron in an RTK and is hypothesized by the authors to play a wider role in other positive feedback loops.

Control of protein stability by post-translational modifications has emerged as an important mechanism for controlling the expression and function of key cellular proteins (reviewed in (7). In particular, RTKs, which are central triggers of cellular mitotic effects in actively proliferating tissues and cancer, have been described to be targeted for ubiquitin-proteasome system (UPS)-mediated degradation by ubiquitin E3 ligases like CBL (4, 8, 9). In fact, human Cbl proteins are activated through tyrosine phosphorylation, thus providing a feedback loop whereby the activation of tyrosine kinases leads to their own degradation (10). In the cases reported so far, the Growth Factor (GF)-triggered signal promoted the *bona fide* degron motif-containing RTK degradation, as part of a negative feedback meant to down-modulate the RTK-signaling activity (3). The presence of a Degron domain in all these cases is based on the demonstration of targeted ubiquitination of the studied RTK via CBL binding and consequent UPS-mediated clearance. The more recent characterization of an autocrine GF signal rescuing (rather than promoting) an oncogenic RTK protein degradation (5, 6) indeed provides a mechanism for the steady-state overexpression of the same RTK at the post-translational level. Notably, this effect was found mediated by a phospho-inhibited (*pi*)Degron embedded around the phosphorylated RTK region (5). In this context, the specialized role of the GF-generated signal was to prevent the RTK’s *pi*Degron recognition by an underlying ubiquitin E3 Ligase (UbE3L)-driven complex, ultimately blocking the RTK UPS-mediated degradation (as graphically summarized in **Figure 2**). The essential molecular components of this regulatory mechanism (see **Figure 2B**), include (a) an RTK bearing a phospho-inhibited (*pi*)Degron domain, and (b) a UbE3L-driven complex docking the *pi*Degron upon growth factor-signal deprivation causing *pi*Degron dephosphorylation. Notably, this mechanism which we here define with the acronym “*Over-Expression by Degradation Rescue*” (*OEDR*) could offer a first mechanistic example of a positive feedback whereby autocrine GF-signals can acutely promote and maintain the overexpression of RTKs in cancer as well as in other RTK-dependent pathophysiological contexts. The realization of the existence of *OEDR*, we believe, can further intensify the search for unknown growth factor-regulated *pi*Degrons within RTKs found expressed in the above contexts. *OEDR* also extends the number of post-transcriptional tools used by a cancer cell in order to maintain its malignant features beyond the known transcriptional, epigenetic and RNA transcript stabilization levels. In light of these evidence-based considerations, it is feasible to envision that specific targeting of autocrine growth factor circuits associated to RTKs’ *OEDR* may disclose a number of therapeutically exploitable anti-cancer effects. It is worth noticing that *OEDR* might similarly affect the expression of other signaling proteins beyond RTKs. However, our present perspective focuses on *OEDR*-regulated RTKs given the pathophysiological implications discussed herein.

## 3 Phospho-regulated Degrons functional differentiation upon pathophysiological context

Another key biological outcome of the functional identification of a phylogenetically conserved phospho-regulated Degron domain, such as the one we identified in the EphB4 RTK (5), relates to the association of this type of Degron with the *OEDR* process. This, is in contrast with previously described phospho-Degron regulated RTKs, in which Degron phosphorylation promoted its protein degradation as previously reported for EGFR, FGFR, PDGFR and TRKA degradation via CBL UbE3L (4, 8, 9). Therefore, based on the aforementioned opposite effects, Degron motifs could be clearly distinguished into either phospho-activated Degrons (“*pa*Degrons”), or phospho-inhibited Degrons (“*pi*Degrons”). Indeed, phospho-regulated Degrons and their role in the regulation of cellular proteins expression has been recognized since a few decades (11, 12). Nonetheless, the report of *bona fide pi*Degrons-regulated proteins to date had been mostly associated to the coordinated expression of cell cycle-related kinases and/or cell-cycle regulated transcription factors (reviewed in (13). While the functional concepts of positive and negative hormones/growth factors feedback loops are well established in clinical and experimental endocrinology (1), the mechanistic aspects of such self-regulatory signals have been elusive. The recent literature indeed provides additional detail suggesting a specific role of *pa*Degrons and *pi*Degrons towards hormone/GF feedbacks regulation. In fact, the identification of the *OEDR* mechanism for the acute regulation of an RTK expression, along with the proposed differential role of phospho-Degrons for the post-translational regulation of RTKs as envisioned herein, allows to repurpose such unexploited protein domains in a specific pathophysiological context (eg in cancer). According to this proposed scenario, the UPS-degradation of RTKs bearing phospho-activating (*pa*) Degrons, such as those mediated by CBL reviewed in (4) fits with the general features of a ligand/receptor negative feedback loop where an excess of a certain GF-generated signal leads to the downregulation of the expression of the underlying signal transducing RTK (3). Conversely, the GF-induced *OEDR* regulation of an RTK through its phospho-inhibited (*pi*)Degron, such as that underlying the IGF-II/IR-EphB4 axis (5), fits with a positive signal feedback, under the specific pathological and cellular context. On a biological standpoint, this mechanism also explains the long described ability of cancer cells to grow under low serum cultural conditions in which the stabilized/over-expressed RTK decreases the cellular dependence on extracellular growth stimuli, enabling the cancer cell to fully activate its downstream proliferative/gain-of-function signals, even in presence of low amounts of self-secreted growth factor. This dual type of control by phospho-regulated Degron-bearing RTKs is summarized in **Figure 2**. The *OEDR*-regulation of RTKs may play a key role, for example, in the early stages of malignant transformation where initial tridimensional cancer tissue growth occurs in absence of a fully formed vascular network, causing poor access to circulating growth factors along with blood vehiculated oxygen and nutrients. Under such circumstances, the cancer cell upregulates the components of the anaerobic metabolic pathway, compensating for the last two types of restrictions, while *OEDR*-regulation of RTKs, by skipping the gene transcription control level, provides the cancer cell with a rapid and efficient to overcome the extracellular GF stimuli requirement, while still maintaining an effective RTK-mediated intracellular signal.

## 4 OEDR regulation of EphB4 by the IGF-II/IR axis in Cancer: first of a new kind?

EphB4 overexpression in solid cancers has been widely described along with its tumor promoting role in malignancy (14). Our initial observation associating cancer IGF-II secretion with EphB4-expression in solid malignancies led us to a search for a cause-effect link between the two co-expressed oncogenic cellular events. The confirmation of a tight functional dependence for EphB4 protein expression in malignant mesothelioma along with a number of cancer cell lines bearing the IGF-II autocrine signal (5) raised the question on the underlying mechanism. This has been addressed, in part, in the study associated to the phospho-inhibited degron discovery in EphB4 (5), and in wider detail in studies published more recently (6, 15). Specifically, the characterization of the acute post-translational upregulation of EphB4 in response to autocrine IGF-II has been demonstrated by our observation that deprivation of the autocrine IGF-II signal in cultured cancer cells using a neutralizing anti-IGF-II antibody caused a marked and rapid decrease in EphB4 total protein levels (5). This is in agreement with a previous study (16) identifying EphB4 among the IGF-II ligand-specific tyrosine phosphorylated downstream targets of the Insulin-receptor isoform A suggesting that direct phosphorylation of EphB4 by IGF-II was directly linked to its increased protein expression. The possibility of a dual checkpoint for EphB4 expression by IGF-II including a potential effect at the gene transcriptional level has further been ruled out by our study in an optimized mouse embryo cellular model transfected with a number of human EphB4 gene promoter reporter constructs bearing differential regulatory elements, and by comparing the acute stimulatory effect of whole serum against the isolated effect of IGF-II in serum-deprived cells carrying the above reporter constructs. This study ultimately confirmed a negligible effect of IGF-II on EphB4 transcriptional activity compared to serum (17), and further strengthened our findings related to the *OEDR* post-translational regulation of this oncogenic RTK. Our search for the tyrosine residue targeted by autocrine IGF-II as suggested by the previously cited study (16) was conducted in an EphB4 overexpressing mesothelioma cell line (MSTO211H). This led us to: (a) the identification of EphB4 last C-terminus amino acid (Tyr987) as the acutely phosphorylated residue, (b) the finding of an inverse relationship between EphB4-Tyr987 phosphorylation status and EphB4 steady-state protein expression (confirmed under Cycloheximide treatment), (c) the observation of inverse relationship between EphB4-Tyr987 phosphorylation and total ubiquitination of the EphB4 last C-terminus 30 aa, and finally (d) that such autocrine signal by IGF-II on EphB4-Tyr987 was dependent on Insulin receptor (IR) isoform A activation but not the IGF1R, supporting contextual different mechanisms of control of EphB4 expression and function by the IGF ligands/RTKs system in cancer. The single and most relevant finding of this study relates on the effective reduction of EphB4 expressing levels through deprivation of the IGF-II signal via block of its extracellular autocrine loop. This strategy, if adopted at the therapeutic level, simplifies the current approach aiming to block EphB4 cancer-promoting effects through inhibitor compounds requiring membrane translocation towards intracellular targeting of the EphB4 RTK activity. Furthermore, since EphB4 promoting effects in cancer have been found to be variably or negligibly dependent on EphrinB2 extracellular binding (18), the block of IGF-II in cancers with confirmed dependence on IGF-II for EphB4 overexpression overcomes the potential resistance to treatment using anti-EphB4 agents targeting the extracellular/ephrinB2 binding portion of EphB4 (19). Similarly, any foreseeable *OEDR*-regulated RTK circuit linked to a specific growth factor autocrine signal may represent an actionable therapeutic target.

## 5 Implication of OEDR for cellular ubiquitin E3 ligases

As for the proposed phospho-regulated Degron distinction in negative feedback-associated (phospho-activated) Degrons versus OEDR-associated (phospho-inhibited) Degrons, under the same frame also UbE3Ls could be sub-classified as negative feedback-associated UbE3Ls versus positive feedback (*OEDR*)-associated UbE3Ls. While CBL has received various supporting evidences on its broad role in mediating degradation of RTKs (ErbB1/EGFR, PDGFR, FGFR2, TrkA/NGFR, Met/HGFR and CSFR1) consistent with growth factors-activated negative feedback signals (4, 8, 9), the only *OEDR*/positive GF feedback-associated UbE3L described to date is the newly discovered DTX3c variant (6, 15) (**Figure 2B**). Therefore, it is likely that a growing number of *OEDR*-associated UbE3Ls and targeted RTKs will appear in the literature similarly to the described regulation of EphB4 by the autocrine IGF-II/IR signal (5, 6), possibly by adopting the experimental approach used to identify such RTK-regulated mechanism along with the underlying key degradation-rescue machinery (20).

## 6 Conclusions and perspectives

Based upon extended revaluation of original findings contributed by the authors (5, 6), and in light of contextual recent advancements in the field of growth factor signal-mediated UPS degradation of receptor tyrosine kinases, the new concept of *Overexpression-by-degradation-Rescue* (*OEDR*) is here introduced for the first time. It is proposed that *OEDR*-regulation of RTKs plays a constitutive and mechanistic role in previously described “hormone positive feedback loops”, under which a cellular self-stimuli provided by a growth factor promotes the upregulation and maintenance of a downstream directly phosphorylated RTK. The extent of *OEDR*-regulation of a cellular RTK by and autocrine growth factor, which bears the opposite function of a hormone/GF-induced negative feedback loop, is still to be clarified outside our published work. This, is partially due to the lack of optimized experimental methods for the identification and study of Degrons domains, which act as key sensors for the positive and negative feedback signals reported by the studies cited herein. Indeed, a novel experimental approach we set up for our discovery, namely a post-translational-modification-enhanced Degron bait pull-down assay (6, 20), potentially simplifies the biochemical steps for the identification of phospho-regulated degron domains within known cellular RTKs along with the parallel identification of underlying degradation complex components. The adoption of this experimental approach has already been instrumental for the discovery and cloning of the novel ubiquitin E3 ligase isoform DTX3c (21) which we found involved in the process we are here referring as *OEDR* (6, 15). We foresee this strategy to facilitate the search and re-evaluation of autocrine growth-factor signals in a variety of cancer cell types, ultimately providing a new layer of potential actionable mechanisms.

## Data availability statement

The original contributions presented in the study are included in the article/supplementary material. Further inquiries can be directed to the corresponding author.

## Author contributions

PS: Conceptualization, Writing-original draft. SW: Writing-review & editing.
